# Gendered time use during COVID-19 among adolescents and young adults in Nairobi, Kenya

**DOI:** 10.1016/j.eclinm.2022.101479

**Published:** 2022-06-04

**Authors:** Anaise Williams, Shannon N. Wood, H.Colleen Stuart, Grace Wamue-Ngare, Mary Thiongo, Peter Gichangi, Bianca Devoto, Michele R. Decker

**Affiliations:** a Department of Population, Family and Reproductive Health, Johns Hopkins Bloomberg School of Public Health, Baltimore, MD, USA; b Bill & Melinda Gates Institute for Population and Reproductive Health, Department of Population, Family and Reproductive Health, Johns Hopkins Bloomberg School of Public Health, Baltimore, MD, USA; c Johns Hopkins Carey Business School, Baltimore, MD, USA; d Department of Sociology, Gender and Development Studies, Kenyatta University, Nairobi, Kenya; e Women's Economic Empowerment Hub, Kenyatta University, Nairobi, Kenya; f International Centre for Reproductive Health-Kenya, Nairobi, Kenya; g Technical University of Mombasa, Mombasa, Kenya; h Department of Public Health and Primary Care, Faculty of Medicine and Health Sciences, Ghent University, Belgium

## Abstract

**Background:**

Gender disparities in time use contribute to poor outcomes in women. Large-scale disruptions can affect time use. The objectives of this study were to characterize time use across the pandemic by gender and to assess how gender associates with 2021-time use, overall and by 2020 economic dependency status.

**Methods:**

A prospective cohort of youth in Nairobi, Kenya, completed phone-based surveys in August-October 2020 and April-May 2021. Time use was characterized at both time points and 1,777 participants with complete time use data at both time points were included in the analysis. 2021-time use was regressed on gender and stratified by 2020 economic dependency status.

**Findings:**

At both time points, significant gender differences in time use found young men with more time on paid work and less time on domestic work [1·6 h; 95% CI: 1·1, 2·2] and [-1·9 h; 95% CI: -1·1, -1·5], respectively; 2021. In adjusted models, the gender differential in unpaid domestic work were significant overall and at all levels of economic dependency (dependent, semi-dependent, independent). The gender differential in paid work was evident among semi-dependent and independent.

**Interpretation:**

Young women spent less time on paid work and more time on domestic duties than male counterparts, consistently across a six-month period during the pandemic, suggesting gendered time poverty. Resulting gendered gaps in earnings can contribute to women's longer-term economic vulnerability.

**Funding:**

This work was supported by the Bill & Melinda Gates Foundation [010481].

**Research in context**

**Evidence before this study**

Within the limited body of research on time use, evidence of gender-differentiated time use has been used to characterize and understand time poverty among women. We used the search terms (“time use” AND “youth” AND “gender” AND “COVID-19 pandemic” AND “low and middle-income countries”). Regarding time use in the COVID-era, a small number of studies, and few in low- and middle-income countries (LMICs), have explored gendered time use in the home during the COVID-19 lockdowns, with evidence of women taking on increased childcare and housework burdens and being more likely to lose or reduce paid work opportunities, though almost all have focused on married couples. No studies have used a prospective cohort of youth to understand adolescent and young adult time use during the COVID-19 pandemic using a gender lens.

**Added value of this study**

This is the first study to the best of our knowledge to use gender analysis to investigate youth time use and trajectories over a six-month period during the COVID-19 pandemic in a LMIC. We find striking gender disparities in time spent on income generation and household tasks that were persistent over a six-month period during the pandemic, echoing a broader body of work that has focused primarily on adults. Our results add to the evidence base on gendered time poverty by clarifying disparities and highlighting domestic labor burdens of young women relative to men, which may have implications for longer term health and economic stability.

**Implications of all evidence available**

Gendered disparities in time use begin early in life and are evident during the COVID-19 pandemic; disruption to time spent on skill development, notably school and paid work, is one of the ways that the pandemic will affect long-term health and economic stability among youth. Our findings speak to the need for social norms change around the “double workday” burden for young women and the need for targeted methods to support gender equitable transitions to adulthood in the post-COVID era. Further, the available evidence highlights the need for systematic, robust time use surveys among youth that employ a gender lens, particularly in low and middle income countries during protracted economic crisis.

Alt-text: Unlabelled box

## Introduction

Gender is a social determinant of health; this social system confers differential levels of power that shape economic and social status.^1^ Gender is distinct from sex in that it refers to locally defined responsibilities, roles, and entitlements associated with being a man or woman.^1^ The gender system structures the behaviors and experiences of all humans, and is largely reflected in wider trends of how different genders participate in economic, political, and domestic spaces. “Gendered time” has been used extensively in the literature and is conceptualized as identified differences between genders in time allocation to different life activities, such as work, childcare, leisure, socializing, or housework.^2^, ^3^, ^4^

Time poverty in relation to gendered time is often characterized by the “double workday” or “second shift” burden for women, characterized by having both domestic and non-domestic labor responsibilties.^5^^,^^6^ Gender norms that construct the “double workday” for women have clear associations with reduced mental health for women. A U.S. study, for instance, found that inequalities in housework contribute to significantly higher rates of depression in women.^7^ Caused by social and cultural norms that sustain gender divisions in labor, time poverty prevents young women from expanding marketable skillsets and other capabilities on par with that of young men. Therefore, how young men and women spend their time is an important component of gendered poverty. Understanding changes in time use is critical for the promotion of women's health and wellbeing across the life course, particularly in low and middle income countries (LMICs).^5^

Time use is increasingly recognized as an indicator of wellbeing.^8^^,^^9^ For youth, daily time use has been used to estimate youth exposure to socialization, learning, and skill-building.^10^ Further, youth time use is predictive of longer-term health and wellbeing outcomes in adulthood, including household income, welfare take-up, mental health, obesity, teenage pregnancy, and early death.^8^^,^^11^ Long-term quality of life, for instance, is negatively associated with time spent on unpaid domestic and care work.^7^ Youth and young adulthood is a critical time for gaining skills for movement towards sustainable economic self-sufficiency. Time use is a helpful way to capture the lifestyles of young people and to highlight groups of youth that may need additional support for skill-building and wellness more broadly.^8^

Daily time use is also a useful way to capture labor and value assignment by gender.^11^ Such measures provide insight into the household economy, rather than simply the labor market.^12^ Time measures have evaluated gender imbalances through large-scale studies such as the Multinational Time Use Study (MTUS)^13^ through the Centre for Time Use Research and the United Nations Statistics Division Time, conducted with children and adults.^14^ Such work finds that women take on three times the amount of unpaid domestic work than men do.^7^ To date, however, existing time use surveys largely focus on high-income countries; overall, less is known about time use, and gender gaps therein, in sub-Saharan Africa and specifically for youth, who may face heightened gender and power imbalances due to younger age and increased economic dependency on others.

The COVID-19 pandemic created economic hardship, new sources of income, and changes in domestic responsibilites.^15^ A recent longitudinal study on time use in Mexico found a 30% decrease in total time spent on schoolwork during the pandemic, as compared to before the pandemic, among youth ages 12-18, with similar findings among boys and girls.^16^ Some work has highlighted that the pandemic increased the gender inequality gap in unpaid labor, in which women are disproportionately burdened.^7^^,^^17^ Other studies find gender inequitable childcare burdens within couples during the pandemic.^18^

Though the exact numbers of pandemic-induced unemployment are currently unavailable, the economic hardship of the pandemic in Nairobi, Kenya is vast.^19^ Starting in March 2020, the Kenyan government suspended all schools, colleges and universities.^20^ Lockdowns were enacted and many organizations enforced compulsory unpaid leave in mid-2020.^19^ Pre-pandemic data found 29% of households in Nairobi to be completely food insecure, with economic shock due to loss of employment as the main driver of food insecurity, highlighting concerning impacts of COVID-induced widespread job loss on households.^21^

Employment data highlights that young workers are more likely to lose their jobs than older workers during economic crisis, as was found in the United States at the beginning of the COVID-19 pandemic.^22^ Young people in Nairobi face a range of vulnerabilities, including access to decent work.^15^^,^^23^ The economic shock of the pandemic likely impacted youth dependency on parents, partners, and other family members. A recent study on youth relationships in Nairobi found that pandemic-induced hardships increased young women's economic dependency on male partners, potentially emphasizing gender stereotypes of men's role as “providers.”^23^ In light of these findings as well as other evidence of disproportionate economic and social impacts on women during past pandemics,^24^, ^25^, ^26^ a gender analysis of the COVID-19 pandemic among youth becomes increasingly relevant.^27^

In a cohort of youth and young adults, we characterize time use inclusive of paid work, schoolwork, and unpaid domestic work at two points in the COVID-19 pandemic, considering gender differences as well as life stage approximated by economic dependency status. Specific objectives are to 1) characterize time use by gender at two pandemic time points and examine changes therein and 2) evaluate how gender relates to 2021 time use, overall and stratified by economic dependency status. Recognizing that the economic shock of the pandemic may have shaped youth dependency status by the time of our survey, we explore the relationship between pandemic-induced household economic shock and youth economic dependence. This is the first study to use a gender lens to investigate youth time use and trajectories over a six-month period during the COVID-19 pandemic.

The two time points in which young people's time use was assessed in Nairobi were August-October 2020 and April-May 2021, an approximate 6-month interval. During the 2020 survey round, COVID-19 cases were reaching low levels after the June and July 2020 peak. During the 2021 survey round, COVID-19 cases were at a spike.

## Methods

### Design panel

The present analysis uses two time points of data collected from the Performance Monitoring for Action (PMA) Agile Youth Respondent-Driven Sampling Survey (YRDSS) cohort. Adolescents and young adults ages 16-26 were recruited via respondent-driven sampling (RDS), in which seeds of non-randomly selected members of the target population initiate peer recruitment chains; RDS sampling weights account for recruitment probability (discussed below). In August-October 2020, 1,217 young men and young women were surveyed. In April-May 2021, 1,177 [97% retention] were recontacted, consented, and surveyed; the analytic sample for the present analysis is restricted to the 1,777 participants with complete time use data at both time points. Trained resident enumerators (REs) implemented survey data collection by phone in English or Swahili, per participant preference, using OpenDataKit (ODK). IRB approval was obtained from the Ethics Review Committee at Kenyatta National Hospital/University of Nairobi and the Institutional Review Boards at Johns Hopkins Bloomberg School of Public Health. More details on the sample and study procedures are outlined elsewhere.^28^ All the participants provided informed consent prior to be enrolled to the study.

### Measures

#### Outcomes

The time use assessment was adapted from the National Statistical Committee Living Standards Measurement Survey;^29^ participants were asked to think about a regular day within the past week and indicate how many hours per day they spent on each of the following categories: unpaid care for children or adults (unpaid caregiving); paid work in the formal or informal/gig economy (paid work); schoolwork/in school (schoolwork; assessed only among those in school at the time of the survey); and cleaning house, preparing meals, or washing clothes (unpaid housework). For the purposes of this study, we combine time spent on caregiving and housework to create an unpaid domestic labor category. Each time use category was analyzed individually on a continuous scale from 0 to 24 h.

#### Independent variables

Gender is the main independent variable. While gender encompasses more categories than the binary form of man/woman, our data is limited to self-report of being a man or a woman. A second independent variable is the latent construct of economic dependency status in 2020 in the form of a categorical variable created through latent class analysis (LCA). LCA categorizes participants probabilistically on the basis of response patterns to the set of indicators believed to be associated with the latent variable,^30^ specifically, economic dependency status. The binary observed variables included in the LCA as measured in 2020 are 1) cohabitation (with parents vs. with partner, self, or other), 2) household prime earner (self vs. other), 3) responsibility for paying for the participants basic needs (self vs. others), and 4) participant responsible for paying for other's basic needs (no vs. yes). The LCA generated three classes that capture youth dependency status: 1) dependents (33%), 2) semi-dependents (39%), and 3) independents (29%; Appendix 1). Dependents are young people who live with their parents and fully depend on others for their basic needs, whereas independents are the primary household earners. Semi-dependents are youth that appear to be transitioning out of dependency on their parents; about half live with parents, half pay for their own basic needs in part, and half pay for other's basic needs at least in part, but only 2% are the primary household earner.

#### COVID-specific measures for contextualization

Pandemic-induced household economic shock is measured as any household member (including the participant) having any disruption to formal or informal income generation due to Covid-19 restrictions, measured in 2020.

#### Covariates

Standard measures characterized age, marital status, and whether the participant has children; these measures, assessed at 2021, were included within final adjusted models.

### Statistical analysis

We first describe time use at each time point (2020, 2021), and examine change in time use across the timepoints, stratified by gender. At each time point, gender differences in time on paid work, schoolwork, and unpaid domestic work were evaluated by weighted means testing. For analysis of the change in time spent on school, the sample was restricted to only those who reported that being a student was their main activity at both time points. Given modest changes in time use across time points, 2021 time use is the outcome for subsequent analyses.

We describe characteristics overall and by gender, and by economic dependency status (2020); differences between genders overall and stratified by economic dependency were assessed via design-based F-statistics. All results are weighted; we also present unweighted sample characteristics (presented in Annex). Following bivariate models (not shown), multivariable linear regression models examined the association of gender with time use by category (paid work, schoolwork, unpaid domestic labor), adjusting for dependency status and other confounders (age, marital status, children). Models were subsequently stratified by dependency status to examine the consistency of disparities identified by life stage.

Post-hoc analysis explored whether the pandemic may have influenced economic dependency status, in lieu of pre-pandemic dependence data. Specifically, we assess correlations between pandemic-induced household economic shock and youth dependency using tetrachoric correlation coefficients for each status independently (binary) and pairwise Pearson's correlation coefficients for status overall (categorical). Percent household shock overall and by economic dependency status, and gender therein, are displayed via bar graph along with statistical significance of the correlation coefficient.

All analyses were conducted using Stata 15.1 (College Station, TX) with statistical analysis a priori at p<0.05. Sampling weights accommodate the RDS study design using RDS-II (Volz-Heckathorn) weights, which account for participant network size as a proxy for likelihood of study recruitment. Post-estimation weighting adjustment was conducted based on comparison of age and educational levels of the sample to the 2014 KHDS population data. Loss to follow-up weights were calculated by regressing the sociodemographic characteristics of participants on their odds of completing the follow-up survey and taking the inverse of the predicted probability from that model. A final weight accounts for the RDS, post-estimation, and loss-to-follow-up. All presented estimates are weighted, and statistical testing accounts for robust standard error clustering by sampling frame and survey design weighting. No missing values exist across outcomes, predictors, and covariates within the analytic sample.

### Role of the funding source

The funding source played no role in the study design; collection, analysis, and interpretation of data; in writing the report; or in the decision to submit the paper for publication. All the authors had access to the data and decided to submit the manuscript for publication.

## Results

At both time points, young men spent significantly more time on paid work and significantly less time on unpaid domestic work relative to young women (2020: 1·9 h_paid work_ [95% CI: 1·4, 2·4]; -2·4 h_domestic work_; [95% CI: -2·9, -2·0]; 2021: 1.6 h_paid work_ [95% CI: 1·1, 2·2]; -1.9 h_domestic work_ [95% CI: -1·1, -1·5]; Table 1). No gender differences in time spent on school were observed among those whose main activity is attending school. Over the six month interval, time spent on schoolwork increased by about one hour for both genders (1·4 h_women_ [95% CI: 0·8, 2·0]; 1.2 h_men_ [95% CI: 0·5, 1·9]), whereas time spent on unpaid domestic work decreased among young women only (-0·7 [95% CI: -1·1, -0·4]).

**Table 1:** Time use in hours per day by gender.

	2020			2021			Difference 2021-2020	
	Young Women Mean Hours	Young Men Mean Hours	Men - Women Estimate (95% CI)	Young Women Mean Hours	Young Men Mean Hours	Men - Women Estimate (95% CI)	Young Women Estimate (95% CI)	Young Men Estimate (95% CI)
Paid work	1.8	3.7	1.9* (1.4, 2.4)	2.2	3.8	1.6* (1.1, 2.2)	0.3 (0.0, 0.6)	0.1 (-0.3, 0.4)
Unpaid domestic work	4.6	2.2	-2.4* (-2.9, -2.0)	3.9	2.0	-1.9* (-1.1, -1.5)	-0.7* (-1.1, -0.4)	-0.2 (-0.4, 0.1)
Schoolwork	1.8	2.0	0.2 (-0.3, 0.7)	3.1	3.4	0.3 (-0.4, 1.0)	1.4*^,^+ (0.8, 2.0)	1.2*+ (0.5, 1.9)

⁎ p-value<0.05; means testing adjusting for weighting and clustering.

+ Sample limited to those who report being a student as their main activity at both time points.

Table 2 outlines sample demographics overall, by dependency status, and gender therein. While young men and women about equally comprised dependents (44% vs. 56%), 76% of independents were men and 64% of semi-dependents were women. Overall, dependents had the least gendered differences in demographics. Among the full sample, main activity differed significantly by gender (p<0·01); within dependency groups, however, significant gendered differences were not observed in main activity. There were no significant gendered differences in age among the full sample. However, women semi-dependents were significantly older than male semi-dependents (p<0·01). There were no significant gendered differences in school enrollment status among the full sample. Among dependents, however, young men were significantly more likely to be enrolled in school than young women (74·9% vs. 53·2%, p<0·01). There were no significant gendered differences in marital status among the full sample. Yet among semi-dependents, young women were significantly more likely to be married than young men (15·4% vs. 2·1%, p<0·01). Young women overall were significantly more likely to have children than young men (32·7% vs. 12·8%, p<0·01). The only dependency group without gendered differences in parenthood was the dependents group.

**Table 2:** Sample characteristics of Nairobi youth and young adults (April-May 2021); by gender, overall and by economic dependency status, weighted percentages

	Overall n=1,177 (100%)			Economic dependency status+								
				Dependents n=385 (33%)			Semi-dependents n=455 (39%)			Independents n=337 (29%)		
	Alln=1,177(100%)	Women n= 591 (50%)	Menn=586 (50%)	Alln=385(100%)	Womenn=217(56%)	Menn=168 (44%)	Alln=455 (100%)	Womenn=293 (64%)	Menn=162 (36%)	Alln=337 (100%)	Womenn=81 (24%)	Menn=256 (76%)
**Main activity**			**<0.01**++			0.48++			0.25++			0.06++
Employed in formal economy	5.0	3.8	6.7	1.5	1.0	2.8	6.9	6.4	8.1	6.5	3.3	7.7
Working informal economy	29.1	26.1	33.3	14.8	16.7	10.0	27.9	27.9	28.2	46.1	47.4	45.6
Student	29.1	31.4	25.9	57.3	53.5	67.3	20.8	19.0	25.3	8.9	8.8	8.9
Caregiver	4.8	6.2	2.9	5.6	5.7	5.3	5.5	7.3	1.0	3.2	4.2	2.8
Self-employed	16.1	13.2	20.1	5.8	6.5	3.8	20.5	19.5	23.1	21.7	11.9	25.4
Other	15.9	19.3	11.1	15.1	16.6	11.0	18.5	20.1	14.3	13.7	24.4	9.7
**Age**			0.59++			0.18++			**<0.01**++			0.24++
16-18 years	15.3	15.8	14.4	36.8	32.7	47.5	8.3	6.0	14.3	0.4	0.0	0.6
19-21 years	31.7	32.9	30.0	40.4	42.5	34.9	33.3	30.0	41.6	20.1	14.7	22.2
22-26 years	53.1	51.3	55.1	22.8	24.9	17.6	58.4	64.1	44.1	79.5	85.3	72.2
**School status**			0.87++			**<0.01**++			0.07++			0.63++
Currently in school	32.4	32.1	32.7	59.2	53.2	74.9	22.6	19.6	30.4	15.3	12.7	16.3
**Married**			0.25++			0.61++			**<0.01**++			0.98++
Married/cohabiting	7.7	8.7	6.3	0.1	0.2	0.1	11.6	15.4	2.1	11.0	11.1	10.9
**Parenthood**			**<0.01**++			0.24++			**<0.01**++			**<0.01**++
Percent with children	24.5	32.7	12.8	10.8	13.0	4.9	32.3	40.1	12.4	29.5	64.6	16.3

+ Characterized by 2020 cohabitation status, prime household earner status, dependency for basic needs, and providing for others’ basic needs through latent class analysis (LCA). Breakdown of factors by status presented in Annex Table 1.

++ P-value of the gendered difference; bolding signifies significance; statistical testing through design-based F statistic adjusting for weighting and clustering.

In adjusted models, being a young woman as compared to a young man was associated with an average 1 h [95% CI: -1·6, -0·5] less time spent on paid work (Table 3). The gendered difference in paid work was greatest among semi-dependents (-1·7 h [95% CI: -2·7, -0·7]) and was also significant among independents (-1·2 h [95% CI: -2·4, -0·1]), though not observed for dependents. Overall, being a young woman was associated with 1·4 h [95% CI: 1·0, 1·8] more time spent on unpaid domestic work; this differential domestic labor burden persisted across all dependency groups and was greatest among independents (1.8 h [95% CI: 1·0, 2·6]). No gender differences were observed in time spent on school among those who report school as a main activity, by dependency or gender, adjusting for other factors.

**Table 3:** Multivariable linear regression of 2021 time use on gender and dependency status, overall and dependency status-stratified, weighted.

	Full Sample	Dependents n=385 (33%)	Semi-dependents n=455 (39%)	Independents n=337 (29%)
	Coeff (95% CI) ^±^	Coeff (95% CI) ^±^	Coeff (95% CI) ^±^	Coeff (95% CI) ±
**Time on paid work**				
Woman	-1.0⁎⁎⁎ (-1.6, -0.5)	-0.2 (-1.0, 0.6)	-1.7⁎⁎⁎ (-2.7, -0.7)	-1.2* (-2.4, -0.1)
Dependents	Ref	–	–	–
Semi-dependents	0.8⁎⁎ (0.2,1.4)	–	–	–
Independents	1.7⁎⁎⁎ (0.9,2.5)	–	–	–
Observations	1177	385	455	337
*Mean hours women*	*2.2*	*1.3*	*2.5*	*3.6*
*Mean hours men*	*3.8*	*1.3*	*3.7*	*4.9*
**Time on domestic work**				
Woman	1.4⁎⁎⁎ (1.0, 1.8)	1.4⁎⁎⁎ (0.6, 2.1)	1.2⁎⁎ (0.4, 1.9)	1.8⁎⁎⁎ (1.0, 2.6)
Dependents	Ref	–	–	–
Semi-dependents	-0.1 (-0.6, 0.4)	–	–	–
Independents	-0.7* (-1.4, -0.1)	–	–	–
Observations	1177	385	455	337
*Mean hours women*	*3.9*	*3.8*	*4.0*	*3.5*
*Mean hours men*	*2.0*	*2.4*	*2.3*	*1.7*
**Time on school**				
Woman	-0.1 (-0.9, 0.6)	-0.1 (-1.1, 0.9)	-0.3 (-1.7, 1.2)	0.6 (-2.1, 3.4)
Dependents	Ref	–	–	–
Semi-dependent	-0.3 (-1.2, 0.6)	–	–	–
Independents	0.7 (-0.6, 1.9)	–	–	–
Observations	331	212	94	25
*Mean hours women*	*3.1*	*3.2*	*2.9*	*2.8*
*Mean hours men*	*3.4*	*3.4*	*3.0*	*3.9*

Linear regression, accounting for robust standard error clustering by node and survey design weighting.

⁎ p<0.05.

⁎⁎ p<0.01.

⁎⁎⁎ p<0.001.

± Models adjust for age, marital status, and whether the participant has children as measured in 2021.

**Figure 1:**
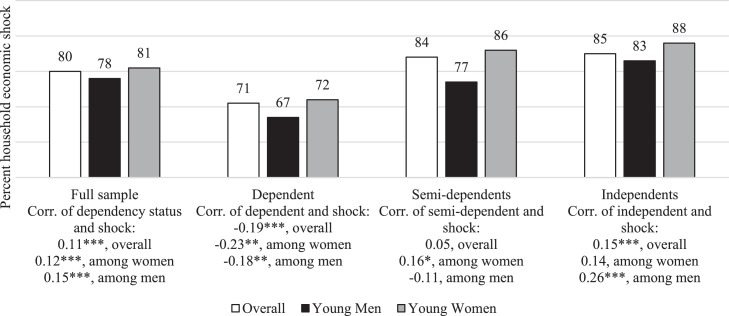
Caption: The figure depicts prevalence of pandemic-induced household economic shock by gender and economic dependency status. The y-axis is percentage of respondents who report their household experienced pandemic-induced economic shock, measured in 2020 as any household member (including the participant) having any disruption to formal or informal income generation due to COVID-19 restrictions. White bars signify overall prevalence, black bars signify prevalence for men, and gray bars signify prevalence for women; bars are separated out by the full sample, dependents only, semi-dependents only, and independents only. Correlations are reported below the bar chart. Using Pearson's correlation coefficient for categorical (full sample) and tetrachoric coefficient for binary (by economic dependency status), we report the correlations between status and household shock among both men and women, among women only, and among men only. P-values for the correlations are noted as: *p<0.05; **p<0.01; ***p<0.001.

Approximately 80% of households had a member (participant or others) who had their income generation affected by the pandemic (Figure 1). Pandemic-induced household economic shock was slightly higher for young women (81%) than young men (78%). Pandemic-induced household economic shock was significantly correlated with youth and young adult economic dependency status (0·11, p<0·001). While the correlation between pandemic-induced household economic shock and being a dependent was negative and significant (-0·19, p<0·001), the correlations between pandemic-induced household economic shock and being an independent earner was positive and significant (0·15, p<0·001). Pandemic-induced household economic shock was highest among independents (85%). Being an independent woman and experiencing pandemic-induced household economic shock was positively correlated but not significant (0·14, p=0·211), whereas being an independent man and experiencing pandemic-induced household economic shock was both positive and significant (0·26, p<0·001).

## Discussion

This first study to apply a gender and life course lens on youth time use during the COVID-19 pandemic finds that time use is highly gendered. Current evidence that young women spend significantly less time on paid work and more time on domestic duties, relative to young men, and these differences remained stable over a six-month period raise significant concerns for gender equity. The gendered domestic labor difference persisted in adjusted models and was evident across dependency levels within the cohort, highlighting stark gender imparity in household labor. Notably, gender differences were not detected in time spent on schoolwork among youth who report school as their main activity; increases in time spent on schoolwork over the six-month interval are promising. The impact of gendered time poverty, and gender gaps in time spent on income generation relative to unpaid household labor, is likely exacerbated by the gender wage gap. These time disparities must be addressed to ensure economic gender equity. This study lacks a pre-pandemic baseline, leaving unanswered questions about the role of the pandemic in the time use disparities identified. Pandemic recovery efforts to rebuild economic stability for youth must consider a gendered lens.

Gender differences in time use varied by life stage, approximated here by economic dependency status. As commonly seen, the transition away from full economic reliance on parents is gendered, with young men far more likely to be economically self-sufficient.^31^ Young men are likely to be either fully dependent on parents or prime earners, with young women far more likely to be semi-dependent (i.e., partial dependency on others and not the prime household earner). Further, pre-pandemic main activity (employee, student, caregiver, etc.) differs significantly by gender overall, yet *within* dependency groups we do not find significant gendered differences, suggesting economic dependency status may account for gendered differences in main activity. Among youth who were dependent on parents six months prior, no gender differences were noted in time spent on schooling and paid labor. Gender disparity in time use increases among young people who became semi or fully independent; for instance, young women who have transitioned to economic independence spend significantly less hours on paid work than their male counterparts and domestic labor appears to substitute for this time. The gender disparities in paid labor are found among youth that have partially or fully transitioned out of parental economic dependency; by contrast, gender differences in domestic labor are persistent across the adolescent life course.

Pandemic-induced household economic shock was correlated with adolescent and young adult economic dependency. This finding is aligned with trends seen in other areas of the world, such as in the US where the Pew Research Center found in 2020 the share of young adults ages 18-29 living with their parents rose to levels not seen since the Great Depression, at around 52%.^32^ While we are not able to confirm temporality, our data suggest youth dependency status in Nairobi approximately five months after the pandemic onset could be partly shaped by the economic disruption of COVID-19. Correspondingly, full parental dependency was negatively correlated with pandemic-induced household economic shock, suggesting that pandemic disruptions were less common among households with parents, or older adults, as the main earners. This group is also where we see the smallest gendered differences in time use. Conversely, being a young male household prime earner was correlated with pandemic-induced household economic shock; it is plausible that these young men took on the economic responsibility for the household when the disruption occurred. Our study is one of the first to link youth economic dependency in the context of widespread economic shock with time use. Future research should more closely assess effects of pandemic disruption on youth economic dependency, given gendered implications for youth time use and skills development.

The study is not without limitations. Despite the prospective nature of the study, we are unable to discern temporality among pandemic-induced household economic shock, dependency status, and time use. We lack a pre-pandemic assessment of time use, thus we are only able to characterize changes *during* the pandemic time points assessed, rather than *since* the pandemic. While time use is ideally measured with multiple methods to measure daily activity at multiple occasions across time and place,^10^ due to survey length constraints, we rely on a single, self-reported measure of time use. Error could be introduced by circumstantial factors near the time of survey assessment, e.g., visiting a family member or on break from school. The economic dependency indicator is limited by ambiguity on whether self-earning was intentionally chosen self-sufficiency or due to not having anyone to depend on, which limits our ability to fully characterize the “independents” group. Results are most generalizable to youth and young adults living in Nairobi, and may not generalize more broadly to rural areas or other LMICs given differences in economic structures and pandemic restrictions. While our study focuses on gender, we note that our data are restricted to only self-identified men and women, and are not inclusive of non-binary individuals. We do not have a measure on ethnicity, and therefore are unable to adjust for ethnicity in our models. Finally, the nature of our study does not inform on *why* youth are spending time the way they are. Qualitative research is needed to explore level of autonomy in time use, time use decision-making and external influences on youth time use both during the pandemic and through the post-pandemic era.

Results highlight the value of systematic, robust time use surveys, with particular attention to unpaid household work and caregiving, using a gender lens and with a focus on youth and young adults. Further research is needed to understand gendered differences in youth transitions to economic self-sufficiency and their impact on economic and health outcomes and disparities later in life. Mapping and addressing gendered time poverty is critical for gender equity efforts broadly, as well as for interventions to prevent disparities and mitigate their impact. Working with young people, particularly men, on equitable domestic labor practices in the home is critical for shifting social norms in the direction of supporting young women's skill development. Sustainable change requires both social norms change and policy that explicitly seeks to redistribute unpaid domestic work across genders.

Understanding gendered differences in time use through adolescent development and transition into young adulthood, in the context of a global disruption, can inform equitable transitions to adulthood in the post-COVID era. Largescale youth jobs initiatives, such as the Kenyan government's Kenya Youth Employment and Opportunities Project (KYEOP), will benefit from gendered analysis to monitor gender equity in enrollment and retention, gendered barriers to participation including domestic responsibilities. Ensuring paid work opportunities and the necessary domestic support for women would help mitigate the impact of widespread household economic shock. Given high participation in the informal labor market for this population, microfinance and workplace travel safety mitigation efforts can substantially support gender equitable access to paid work. For those in the formal labor market, paid leave and subsidies for people with caregiving responsibilities are further measures that would help reduce gendered poverty. Finally, raising adolescent and young adult voices through taskforces and post-pandemic planning is critical; targeted efforts can help minimize gender disparity in longer-term skill development that can be sustained in the post-COVID-19 era.

## Contributors

Anaise Williams: literature search, data analysis, data interpretation, writing

Shannon N. Wood: literature search, data analysis, data interpretation, writing

H. Colleen Stuart: study design, data interpretation, writing

Grace Wamue-Ngare: study design, data interpretation, writing

Mary Thiongo: study design, data collection, data interpretation, writing

Peter Gichangi: study design, data collection, data interpretation, writing

Bianca Devoto: study design, data interpretation, writing

Michele R. Decker: study design, data interpretation, writing, funding acquisition

All authors read and approved the final version of this manuscript.

## Declaration of interests

None to declare.

## Data sharing statement

Data are available by request at pmadata.org and upon reasonable request to the corresponding author.

## References

[1] Heise L., Greene M.E., Opper N. (2019). Gender inequality and restrictive gender norms: framing the challenges to health. Lancet North Am Ed.

[2] Beck M.E., Arnold J.E. (2009). Gendered time use at home: an ethnographic examination of leisure time in middle-class families. Leisure Studies.

[3] Sirianni C., Negrey C. (2000). Working time as gendered time. Feminist Economics.

[4] Woodward A., Lyon D. (2000). Gendering Elites.

[5] Blackden M., Wodon Q. Gender, time use, and poverty: introduction. 2006. Published in: Gender, Time Use, and Poverty in sub-Saharan Africa (edited by Mark Blackden and Quentin Wodon, World Bank Working Paper), 1-10

[6] Hochschild A. The second shift: working parents and the revolution at home New York: Viking Penguin Inc. 1989.

[7] Seedat S., Rondon M. (2021). Women’s wellbeing and the burden of unpaid work. BMJ.

[8] Hunt E., McKay EA. (2015). What can be learned from adolescent time diary research. J Adolesc Health.

[9] Ferrar K., Chang C., Li M., Olds TS. (2013). Adolescent time use clusters: a systematic review. J Adolesc Health.

[10] Lam C.B., McHale S.M. (2015). Time use as cause and consequence of youth development. Child Dev Perspect.

[11] Golsteyn B.H., Grönqvist H., Lindahl L. (2014). Adolescent time preferences predict lifetime outcomes. Econ J.

[12] Apps P. (2003).

[13] Fisher K., Gershuny J., Gauthier A. (2012).

[14] UN (2018).

[15] Banati P., Jones N., Youssef S. (2020). Intersecting vulnerabilities: The impacts of COVID-19 on the psycho-emotional lives of young people in low-and middle-income countries. Eur J Dev Res.

[16] Boruchowicz C., Parker S.W., Robbins L. (2022). Time use of youth during a pandemic: evidence from Mexico. World Development.

[17] Chauhan P. (2021). Gendering COVID-19: impact of the pandemic on women’s burden of unpaid work in India. Gender Issues.

[18] Sevilla A., Smith S. (2020). Baby steps: the gender division of childcare during the COVID-19 pandemic. Oxford Rev Econ Policy.

[19] Suleiman MA. (2020).

[20] Aluga MA. (2020). Coronavirus disease 2019 (COVID-19) in Kenya: preparedness, response and transmissibility. J Microbiol Immunol Infect.

[21] Onyango E.O., Crush J., Owuor S. (2021). Preparing for COVID-19: Household food insecurity and vulnerability to shocks in Nairobi, Kenya. PLoS One.

[22] Economic policy institute current population survey extracts. 2020;1.0.9. https://www.epi.org/publication/young-workers-covid-recession/

[23] Karp C., Moreau C., Sheehy G. (2021). Youth relationships in the era of COVID-19: a mixed-methods study among adolescent girls and young women in Kenya. J Adolesc Health.

[24] Davies S.E., Bennett B. (2016). A gendered human rights analysis of Ebola and Zika: locating gender in global health emergencies. Int Aff.

[25] Wenham C., Smith J., Davies S.E. (2020). https://www.nature.com/articles/d41586-020-02006-z.

[26] WHO (2007). https://apps.who.int/iris/bitstream/handle/10665/43644/9789241595346_eng.pdf.

[27] Wenham C., Smith J., Morgan R. (2020). COVID-19: the gendered impacts of the outbreak. Lancet.

[28] Decker M.R., Wood S.N., Thiongo M. (2021). Gendered health, economic, social and safety impact of COVID-19 on adolescents and young adults in Nairobi, Kenya. PLoS One.

[29] (NATSTATCOM) NSC. Living standards measurement survey the Republic of Kyrgyzstan household questionnaire. 1998.

[30] Hagenaars JA, McCutcheon AL. (2002).

[31] Musau B.M. (2012). http://erepository.uonbi.ac.ke:8080/xmlui/handle/123456789/8163.

[32] Fry R., Passel J., Cohn D. (2020).

